# Shift work to balance everyday life - a salutogenic nursing perspective in home help service in Sweden

**DOI:** 10.1186/s12912-014-0054-6

**Published:** 2015-01-13

**Authors:** Madelaine Törnquist Agosti, Ingemar Andersson, Göran Ejlertsson, Ann-Christin Janlöv

**Affiliations:** School of Health and Society, Kristianstad University, 291 88 Kristianstad, Sweden; Department of Clinical Sciences, Malmö, Lund University, Malmö, Sweden

**Keywords:** Salutogenesis, Nursing, Home help service, Health promotion, Work-life balance

## Abstract

**Background:**

Nurses in Sweden have a high absence due to illness and many retire before the age of sixty. Factors at work as well as in private life may contribute to health problems. To maintain a healthy work–force there is a need for actions on work-life balance in a salutogenic perspective. The aim of this study was to explore perceptions of resources in everyday life to balance work and private life among nurses in home help service.

**Methods:**

Thirteen semi-structured individual interviews and two focus group interviews were conducted with home help service nurses in Sweden. A qualitative content analysis was used for the analyses.

**Result:**

In the analyses, six themes of perceptions of recourses in everyday life emerged;

(i) Reflecting on life. (ii) Being healthy and taking care of yourself. (iii) Having a meaningful job and a supportive work climate. (iv) Working shifts and part time. (v) Having a family and a supporting network. (vi) Making your home your castle.

**Conclusions:**

The result points out the complexity of work-life balance and support that the need for nurses to balance everyday life differs during different phases and transitions in life. In this salutogenic study, the result differs from studies with a pathogenic approach. Shift work and part time work were seen as two resources that contributed to flexibility and a prerequisite to work-life balance. To have time and energy for both private life and work was seen as essential. To reflect on and discuss life gave inner strength to set boundaries and to prioritize both in private life and in work life. Managers in nursing contexts have a great challenge to maintain and strengthen resources which enhance the work-life balance and health of nurses. Salutogenic research is needed to gain an understanding of resources that enhance work-life balance and health in nursing contexts.

## Background

Work-life balance has become a major public issue. This could be related to feelings of job insecurity, more work being done at odd hours, the spread of new information, communication technologies and work intensification [1,2]. The intensification of work reduces time and energy for other activities and has a negative effect on people’s well-being [3]. Besides the changes of work, there are the growing demands of family obligations, a trend due largely to a continued increase in female labor-force participation and that children’s schedules have become more demanding [4,5]. Work-family balance is defined as “a global assessment that work and family resources are sufficient to meet work and family demands to such an extent that participation is effective in both domains” ([6], p.825). In this study, the definition is broadened to *work- life balance* because it makes it possible to find resources outside the family domain. Moreover, studies have shown that the work-family/life interaction impacts more on women than on men in both directions of influence, family-to-work and work-to-family [7]. Women are responsible for maintaining smooth transitions between the worlds of home and work life during the course of life [8].

A critical review of work-life balance research shows that the literature is dominated by studies employing a ‘conflict’ approach, with few using a ‘balance’ perspective [9]. Moreover, there is a lack of qualitative research on employees’ views on work-life balance [10,11]. It is essential to break away from the traditional focus on work-family conflict to a better understanding of new ways to achieve a health-promoting workplace [12]. The European Community Health Promotion Indicator Development Model (EUHPID) demonstrates that health development can be analyzed from a salutogenic (health resources and positive health) and a pathogenic (risk factors and disease) perspective [13]. A majority of the studies of health and ill health has a pathogenic perspective [14] but the salutogenic perspective with focus on positive resources that promote health has been shown to be important in occupational health [15]. Furthermore the salutogenic theory is an important contribution to health promotion research and practice as the salutogenic approach focuses on health promoting processes [16,17]. To understand the determinants for health we need to look for other classes of determinants than the determinants of ill health [18] as focusing on health will be cost and life saving [19]. Researchers highlight the need to focus on the positive consequences of multiple role occupation and how such positive consequences can be achieved [2]. Managers need to actively acknowledge the strong moral and economic grounds that support the development of a healthy workforce [20] and workplace health promotion. Workplace health promotion is workplace initiatives aiming to promote employees’ health from a salutogenic perspective [21].

With increasing economic demands in the society, shift work remains common and adds to the growing complexities of balancing work and family time [4]. Shift work refers to a job schedule in which working hours are other than standard hours of 8 a.m. to 5 p.m. or a schedule other than the standard working week, Monday to Friday [22].

There is a need to focus on the costs and benefits of working non-standard schedules, such as shift work, why people work these schedules and how it impacts their life [23]. Shift work is a common way of working for nurses in home help service in Sweden. Studies of work in care for the elderly have shown that nurses experience their work as meaningful but the disadvantages are that it is stressful, physically and mentally strenuous work with low wages. Nurses in care for the elderly in Sweden perceive high levels of time pressure [24] and have high absence due to illness, and many nurses retire before the age of sixty [25]. Appropriate health-promoting workplace interventions in nursing contexts that ensure the good health of nursing workforce will support, retain and recruit nurses and maintain high levels of care [24,26]. To support employees’ work- life balance is beneficial. When employees are able to combine work and private life, it enhances well-being and health and contributes to a healthy and high performing workforce [27].

An effective workplace health promotion requires an understanding of health and the determinants of health [14]. The EUHPID has been developed to monitor health promotion strategies and highlight the need for systematic salutogenic indicator development for strengthening the health promotion perspective [13]. A qualitative study with a salutogenic approach in a work-life framework intends to give further understanding of work-life balance and strategies in workplace health promotion.

### Aim

This study aimed to explore perceptions of resources in everyday life to balance work and private life among home help service nurses.

## Methods

### Design

A qualitative approach was chosen since we were interested in the nurses’ perception of resources to balance everyday life [28,29]. The methods used for gathering data were individual interviews and focus group interviews. We used different methods, triangulation, to achieve multi-faceted information and to get a more comprehensive picture of the results [30]. The individual interviews contributed to a deeper understanding of the phenomenon and the focus group interviews contributed to a variety of perspectives [29].

### Setting and context

Sweden has a growing proportion of elderly people, seen over a 20 year perspective; one Swede out of four will be over 65. Care for the elderly has therefore become increasingly important. Staff working in home help service in Sweden is assistant and registered nurses. The assistant nurses are mainly licensed practical nurses and nurse’s aides. The licensed practical nurses have three years upper secondary schooling with a focus on care and social services, the nurse’s aides have limited or no basic education in caring. The assistant nurses provide the basic care and everyday domestic work tasks [31] for the elderly people with assistance from municipal-funded home help service. The registered nurses have a three years university education in nursing and are in charge of the medical and specialized nursing care.

One of the aims of the care for the elderly in Sweden is to help these people and those with disabilities live a normal and independent live. To make this possible, elderly people get various kinds of support to make life easier. Support from home help service are prerequisites for elderly people to live in their own homes as long as possible. When an elderly person needs home help service, he or she can apply for assistance from municipal-funded home help service. Each municipality decides its own rate for care for the elderly and the cost depends on the help provided and the person’s income. Elderly people with disabilities can receive both healthcare and social care in their homes around the clock [32].

The participants in this study were registered and assistant nurses working in care for the elderly, home help service, in a municipality in the south of Sweden. In the care for the elderly department in the municipality, there were about 280 employed nurses, 250 assistant nurses and 30 registered nurses. Most of the nurses worked a day and evening schedule, with working times between 7 a.m. to 10 p.m. and every second weekend. They worked on a morning shift or an evening shift. The nurses working on a day and evening schedule had influence over what shifts and days to work through the computer scheduling program *Time Care*. The nurses working on night shifts were working from 10 p.m. to 7 a.m. and they worked every third weekend. The employment for nurses on night shift was 66 percent and was equivalent to a day and evening employment on 75 percent pay. A large majority of the nurses’ working day and evening, in the municipality worked part time, 75–85 percent; 100 percent employment is 38 hours a week.

### Procedure

**Figure 1:**
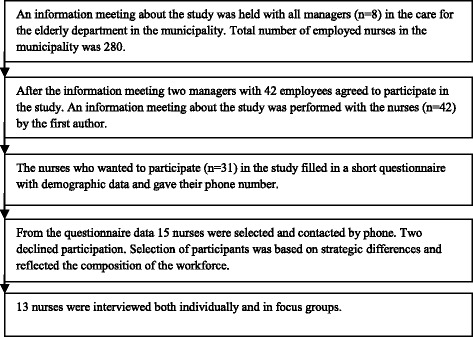
**Flow chart for sample selection.**

An information meeting about the study was conducted with all managers (n = 8) of the care for the elderly departments in the municipality. The two participating home help service departments declared their interest in participation in the study. One home help service department where the nurses worked day and evening schedules (n = 30) and one department where the nurses worked night shifts (n = 12) were offered to participate in the study (Figure 1).

To capture the nurses’ interest in participation in the study, information meetings were held in their workplaces. The nurses who wanted to participate completed a short questionnaire with demographic and family data. Of the 42 nurses (38 assistant nurses and four registered nurses) in total, 31 chose to complete the questionnaire (30 assistant nurses and one registered nurse) and from the answers 15 were selected strategically and contacted by phone. Two declined participation. Selection of participants was based on strategic differences and reflected the composition of the workforce. The purpose of choosing participants based on differences was to explore the work-life balance from different perspectives and phases of life. The 13 participants were invited to a further information meeting and time for an interview was booked. The individual interviews and focus group interviews were conducted in a conference room in a building near the workplace.

### Participants

The participants were women, 12 assistant nurses and one registered nurse. Of the assistant nurses ten were licensed practical nurses and two were nurse’s aides. Their ages ranged from 23 to 57 years, eight were married and five were single. Ten participants had children of different ages, from pre-school children or teenagers to adults. Three of the participants were immigrants while the others had a Swedish background. Three participants worked full time and ten worked part time. Ten participants were working shifts between 7 a.m. to 10 p.m. and every second weekend. They worked on a morning shift or an evening shift, and the number of hours worked each shift depended on whether they worked part time or full time. One of the participants only worked during daytime, but had previous experience of working shifts. Two participants were only working nights, and their schedules were from 10 p.m. to 7 a.m. and they worked every third weekend and part time.

### Ethical considerations

The study was conducted in agreement with the Swedish Law of Research Ethics, SFS 2003:460 as advised by the Local Ethical Review Board of Lund. Participants were informed that their participation was voluntary and that they had a right to withdraw from the study at any time. Before the interview, the participants gave their written informed consent to participate. They were also informed that confidentiality would be preserved. This study was a part of a comprehensive PhD thesis work, ethically approved by the Local Ethical Review Board of Lund and funded by Kristianstad University.

### Data collection

The 13 individual interviews were conducted in 2010 and the two focus group interviews were conducted in 2011.

#### Individual interviews

The individual interviews were based on a semi-structured interview guide [29]. A pilot interview with one assistant nurse tested the interview guide. No changes were made and this interview was included in the analysis. The interviews were in the form of an everyday dialogue constructed between the participant and the researcher [33]. All interviews were conducted by MTA. The interview questions had a salutogenic approach and focused on health promoting factors such as “What recourses in your everyday life are essential to your work-life balance?”. “When does work and private life interact in a positive way?” etc. One interview lasted 45 minutes and the others between 1, 5 – 2 hours. All interviews were recorded digitally and transcribed verbatim.

#### Focus group interviews

The focus group interviews were conducted with the same participants as the individual interviews. There were six participants in the first group and seven in the second group, together with one moderator, MTA, and one observer, A-CJ. The purpose of the focus group interviews was twofold: firstly, to involve the participants in the analysis to confirm that the participants recognise themselves in the data; secondly, the focus group interviews contributed to more data about the phenomenon explored in the interviews. In the focus group interviews, the participants could hear their colleagues’ responses and they could make additional comments beyond their own original responses, and these comments contributed to a wider understanding of the phenomenon [34]. In the focus group interviews, the participants discussed the preliminary themes that emerged from the analysis of the individual interviews. The focus group interviews lasted for two hours. The focus group interviews took place in the same conference room as the individual interviews and were recorded digitally and transcribed verbatim. Unfortunately, the recorder did not function in the second focus group, so the core of the contents of this interview was written down afterwards. A group interview was conducted with two of the participant in the second focus group. This made it possible to get further data about the work-life balance phenomenon and to confirm that the contents that was written down afterwards was in accordance with the participants’ views. The group interview was recorded digitally and transcribed verbatim and numbered 2b in the result.

### Analysis

To answer the research question, a qualitative content analysis was used. The themes have flowed from the data, and were not predefined, to allow new insights to emerge. The analysis started with the authors (MTA, IA, A-CJ) reading the individual interviews separately several times to provide an overall sense of the material. All authors wrote down a naive understanding of the participants’ perception of work-life balance as a whole. The naive understandings were then reflected on and discussed and brought together to one. The data from the individual interviews was then analysed according to Graneheim and Lundman [35] recommendations. The texts about the participants’ perceptions of resources in everyday life to balance work and private life were brought together into one text, which constituted the unit of analysis. Then meaning units were identified, condensed, abstracted and labelled with codes. Through identification of differences and similarities between the codes, further abstraction could be achieved. Twelve sub-themes emerged; the sub–themes were brought together into six themes. The process of the analysis, at each step, was discussed by authors MTA, IA and A-CJ. An example of the analysis from the text is shown in Table 1.

**Table 1:** **Example of categorization procedure**

**Examples of meaning units**	**Condensed meaning unit – description**	**Condensed meaning unit - interpretation**	**Sub-theme**	**Theme**
I did this (took night shifts) mostly because of the children; it was easier when I did not have to leave them in daycare so much/…/ it (working hours) works very well for me and my family (13)	Working nights so the children do not have to be at daycare so much and it works well for me and the family	Working nights is a conscious choice to be there as a mother		
No, then (not working nights) I would not have had time, I am just saying this, my parents and my grandchildren and just being all my life it would never have worked if I had been working Monday – Friday (8)	If I had been working during the day, I would not have had enough time and my life had not worked out so well	Working nights enables relationships and recovery		
It is best that I work evenings and I can be home on my own (in the day) and do exactly what I want, and how I want it without consideration for anybody (FG1)	Working evenings means to be able to be on my own in the day and do exactly what I want	Irregular working hours gives freedom and time for yourself	Working shifts to combine private life and working life	Working shifts and part-time
…when working like this (irregular hours) he must also take responsibility, pack daycare bags and such things, and cook (1)	He must take more responsibility at home when she works irregular hours	Irregular working hours contributes to equality		
I feel that working 75% is enough/…/ you give so much of yourself in this job, I think it is important to have a life at home and a family, now is the time for that (9)	Working 75% is enough, give much of yourself in this job. Important to have a life at home	Part-time work gives time to take care of yourself and have time for both domains	Working less gives more private time	

To increase the validity of the research, two focus group interviews were conducted with the same participants as the individual interviews. The focus group interviews focused on the “preliminary” sub-themes and themes that emerged from the analysis of the interviews. There were no disagreements on the themes as the participants recognised themselves in the result. The focus group interviews gave a wider understanding of the phenomenon. As the focus group interviews did not differ in the perceptions of resources, texts from the focus group interviews were included in the same analysis as the individual interviews.

The researchers’ pre-understanding is part of the interpretation process and was discussed throughout the analysis to guard against researcher bias [36]. The first author (MTA) lives in a dual earning family with pre-school children and has been working as an assistant nurse and with workplace health promotion in a nursing context. IA has many years’ experience of healthcare as a family doctor and works with research in workplace health. A-CJ has a professional experience as a registered nurse, mental health nurse and midwife, with research experience within care for the elderly and psychiatric care and service.

## Results

### Naive understanding

The first naive understanding of the text revealed that when the women were asked about the work-life balance, some found it difficult to describe it, while it seemed natural to others. Familiarity with thinking in terms of a work-life balance seemed to have to do with former life experience, reflections and being interested in keeping a healthy life style. The work-life balance was discussed as puzzle pieces that had to be put together. The work-life balance could be described in terms of two domains, the private life and the work life, which concerned themselves, the family and next of kin, the home and the work. The women could clearly see how the work life and the private life influenced each other in different ways. It was seen as a resource to have both domains in life, as it involves different roles that contribute to meaningfulness, well-being and safety. The women talked about their past, present and future and described that the work-life balance changed at different phases and transitions in life, for example when you have a child or change work. Thus, the work-life balance had to be flexible due to needs of having to reconstruct it depending on the life situation. Values, goals and possibilities in life influenced how much *time* and *energy* the women spent in each domain.

### The structure of the themes

Six themes with sub-themes emerged from the analysis (Table 2). Quotes were numbered according to each interview (1–13) and focus group interview (FG 1-2a and 2b).

**Table 2:** **The interpretation of nurses’ perceptions of resources, sub-themes and themes**

**Sub-themes**	**Themes**
Reflecting over the meaning of life to set goals, boundaries and prioritize	Reflecting on life
Being optimistic to get energy	
Being healthy to manage work and private life	Being healthy and taking care of yourself
Taking care of yourself through health-promoting activities	
Having a job and a supportive work climate gives energy	Having a meaningful job and a supportive work climate
Working with elderly people gives meaning	
Working shifts to combine private life and working life	Working shifts and part time
Working less gives more private time	
Supporting family and next of kin makes the work-life balance easier	Having a family and supporting network
Well-being and health of family and next kin	
Adjusting the home environment to the family needs	Making your home you castle
Decorating and caring for your home makes it a place for well-being	

### Reflecting on life

The text reveals that the participants highlight personal resources such as optimism, meaning-making and life experience as a key resource in everyday life. It seemed important how they thought and what they felt about their lives. Their thoughts influenced how they experienced their work-life balance.

*Reflecting over the meaning of life to set goals, boundaries and prioritize.* Life experiences and different phases in life gave a natural space to reflect on life. When reflecting on life, it becomes easier to prioritize activities of importance and live a life according to one’s own values and goals. To be safe with oneself and have a good self-efficacy helps to not turn yourself inside out in order to please others. To take time to reflect and prioritize in life on a regular basis, and talk about life with others, gave the participants tools to better life management and to personal growth.

*‘You have to get a feel for who you are and take time to think and maybe read books about other people, chat with friends/…/ talk about life and how you do things, etc., and have lots of ideas, and then you take to your heart what suits you and this shapes you to be who you are’ .* (FG 2b)

*Being optimistic to get energy.* It seemed that positive thinking was an important resource to gain energy and to balance everyday life. Thoughts influence attitudes and feelings towards something or someone. Being optimistic about challenges and changes gives energy, instead of draining energy.

‘*Well, thoughts are very important/…/ (before) I had such negative thoughts, but when I wake up nowadays, the first thing I think is "wow I am on my feet, I am healthy", and this is good, I feel joy inside all the time (11)*

### Being healthy and taking care of yourself

It was important to the participants to be healthy and to take care of themselves in order to be able to take care of their jobs and families. Regular activities, such as exercising, contribute to recovery and energy in everyday life.

*Being healthy to manage work and private life.* To stay healthy seemed essential; to have knowledge about your body and recognize body signals, to know when it was time to slow down, set boundaries and to repower. When feeling well and thriving at work, the positive feeling spread to the colleagues and it made the job flow better. A good day at work also had a positive spillover to your private life, in the form of positive feelings.

*It is very important that we feel well and like it on the job, and that will rub off on others as well when you meet a patient, it is a chain reaction’ . (11)*

*Taking care of yourself through health-promoting activities.* It seemed important to spend time in nature. In nature, the participants healed, repowered and were able to distance themselves from a hectic work or family situation. To exercise, read, watch movies, listen to music and go to concerts added quality to life and gave an opportunity to let go of worries and regain energy. To be creative and make things by hand seemed relaxing and contributed to mindfulness and were used like some sort of therapy.

…*and if you are in such a period that you only want to listen to classical music, I also like that, so much is happening in my life, and classical music makes me feel "yes, I can breathe out and be calm"’.* (10)

### Having a meaningful job and a supportive work climate

A meaningful job and a supportive work climate contributed to feelings of safety and well-being. To have a job was important to not being dependent and vulnerable. To have a “second world” to go to when there was trouble at home, to be able to repower and distance yourself and then manage your private life in a better way.

*Having a job and supportive work climate gives energy.* It feels safe to have a job in different ways, financially, a place where you belong and a social network. Supportive co-workers and managers are important resources in life and contribute to a positive work climate, positive energy and thriving at work. The physical environment also plays a role in a supportive work climate. It is essential that there is a physical environment that gives possibilities to interaction with co-workers and relaxation. It seems that a job with a positive work climate can contribute to many positive feelings that spill over to the private domain. A job can be the place where one can recover, heal and get new energy to manage problems at home.

‘*He (my husband) beat me so horribly /…/ if I had not had my job at that time, I would probably not have survived /…/ the job of working with elderly people somehow healed me’ .* (8)

*Working with elderly people gives meaning.* Working with elderly people was described as a meaningful work. A job contributing to feelings of satisfaction, confirmation, to be needed, appreciated and to work with something of importance; to help and give meaning to another person’s life. To work with elderly people also gave thoughts about life and how to live it yourself.

‘*Working with the elderly has given me the idea to take care of my life. When you work with the elderly, you know and you see where you will be at a later stage of your life’.* (FG 1)

### Working shifts and part-time

Working shifts was spoken about in a positive sense to enable a combination of different roles. For some participants, it could be a conscious choice. They could see many advantages with their working schedule and would not want to work differently. To work part-time seemed important to having enough time and energy to other parts of life than work.

*Working shifts to combine private life and working life.* Shift work was spoken of in a positive way and sometimes the only way to combine different roles in life. Shift work gave possibilities of spending more time with one’s children and less time for them in daycare. More free time for the participants to spend alone, for example when they had a day off in the middle of the week, and they did not have to adjust to anyone or anything. Furthermore, shift work gave possibilities to go to the dentist etc. without taking vacation. Shift work contributes to more equality in the home and childcare, when the mother was at work, the father had to take his share of the household. When one or two parents work shifts, one person is often at home, which contributes to fewer days away from work because the children are sick. Furthermore, the job gets more varied through the different routines mornings, evenings and weekends. To work shifts was also described as a way of life (quotes see Table 1).

*Working less gives more private time.* It seemed that working part-time was a strategy for the participants to combine the different roles they had in life. It seemed difficult to work full time and take care of family responsibilities, have time for yourself and next of kin, the home and having an active life outside work and home. The work was also described as demanding, both mentally and physically. When you work part time, your financial resources are less, but material things are of less value. One does not have to earn a lot of money, just enough to get by.

‘*Well, money is not everything, life and being healthy is so much more important, I have enough and that is good enough for me’ .* (FG 1)

### Having a family and a supporting network

To have a family and next of kin gave meaning to life and was spoken of with love and appreciation. The well-being of family members and next of kin influenced the women’s well-being and vice versa.

*Supporting family and next of kin makes the work-life balance easier.* The women spoke of the importance of having a supporting network around them; it made them feel safe, well and gave energy. Furthermore, to have a supporting spouse who listens and has an understanding, makes the work-life balance easier. To spend time with family and next of kin seemed essential to well-being. The women said that children and family gave energy and meaning to life, which strengthened them to manage difficulties.

‘*The very safety of knowing that I have lots of people around me with whom I want to spend time, and should something happen, I know I can call them at any time and they will come over’.* (FG 2b)

*Well-being and health of family and next of kin.* It seems important to see life as a web or a system where you are influenced by others. The participants’ well-being was influenced by family or/and next of kin’s well-being. Co-workers and managers were included in the system and co-workers were also called next of kin. To strive for the health of those around you seems essential.

### Making your home your castle

The home and the home environment are essential to well-being. It was important for the participants to have a home that they liked, thrived in and that lived up to your or/and your family’s needs.

*Adjusting the home environment to the family needs.* To have a space for peace and quiet in the home gave opportunities to reflection, relaxation and distancing. To not live crowded and have space for each family member causes less conflicts and stress at home. The home can also be seen as the world where you are in control, where you can be safe and find your strength.

‘*My home is my world, and nobody disturbs me there, this is where I can regain my (inner) balance’ .*(10)

*Decorating and caring for your home makes it a place for well-being.* To decorate the home was seen as something that gave joy and recovery. When the home was a mess, it contributed to stress and a bad mood that affected family members. It was deemed important that the house was clean and nice. A clean house gave feelings of satisfaction, energy and peace of mind.

## Discussion

The aim of this study was to explore perceptions of resources in everyday life to balance work and private life among home help service nurses. The result indicates that it is of importance to strengthen both individual and organizational resources to promote work-life balance.

### Findings

The findings of this study showed that *shift work* was a resource in everyday life and was used as a strategy to balance work and private life. This result is partly contradictory to most of the literature that has highlighted the negative effects of shift work, particularly concerning health consequences [37,38], but also work-life conflict [39-42]. This unexpected result may partly be explained by the salutogenic approach of this study. By turning the health issue around and looking at what brings health and what recourses are central to balance everyday life, instead of what causes disease and conflicts, we are given different answers, different solutions and are able to identify resources of health [43]. Furthermore, Wayne et al. [2] conclude that conflict and facilitation are orthogonal constructs; programs that have been used for reducing conflict may not effectively increase facilitation. Relying only on what is known about conflict to make practical recommendations is insufficient. Balance may be a more important target for intervention than the traditional conflict measures [44].

The experience of work-life balance may also depend on how the working schedules and working times are constructed. The result showed that the variation between evening and morning shifts was experienced in a positive way. Other studies have shown different result than ours. For example, hours worked during the day or at night gave a similar effect on parental time, but hours worked in the evening showed a much larger negative impact on parental time [45]. Mills and Täht [46] highlight that the lack of negative effects in their study about partnership and non-standard schedules suggests that previous findings – largely U.S. ones – are not universal. In Western Europe, there is higher protection of workers, higher wages and higher benefits. When organizations focus on interventions on social support, team climate and increased control in the work environment the negative effects of shift work can diminish [47,48].

To have the opportunity to work *part time* was also seen as a resource in everyday life. The work was spoken of with meaningfulness, but also physically and mentally exhausting, and time for recovery seemed essential. Part-time work offers more opportunities to slow down [25] and to have *energy* and *time* to spend in other roles in life. Multiple roles are in general beneficial for the individual and the individual’s health, but are dependent on the number of roles and the time demands of them. Just as multiple roles provide opportunities for success, they also offer risks for failure [49]. Wayne et al. [2] highlight the need to focus on the positive consequences of multiple roles and how such positive consequences can be attained. Flexibility has been shown to be a key element that provides opportunities for success for multiple roles [50] and flexible working hours and the opportunity to work part time seem to promote work-life balance and well-being of employees [51,27]. In this study, *working shift and part time* seems to create that flexibility to combine work and private life.

Skinner et al. [42] argue for flexible scheduling and the availability of part-time employment especially for older workers to support their health and well-being. However, a problem part-time nurses may experience in their organizations is limited access to information, advancement opportunities and inflexible scheduling of meetings and education [52]. Another problem part-time nurses may experience is inferior pension possibilities [53]. Therefore is important to work-life balance and to healthcare organizations that the organization strives to integrate part-time nurses to permit more successful achievement of each individual [54].

*Having a meaningful job and a supportive work climate* contributes to energy, positivity and meaningfulness in life. By identifying work-related resources, the employees’ positive experiences at work and the ability to benefit from positive work-related situations may increase [55]. The participants in this study describe the positive spillover from positive work-related situations to private life. To feel meaning in your work, to make a difference for someone else seemed essential. Meaningful work has the power to encourage individual growth [56] and promote health [17]. When work-life conflicts occur, employees tend to find less meaning in their work [56]. Munn [56] concludes that it is essential to consider what creates meaningful work for individuals, how that meaningful work impacts your work-life balance and how work-life balance may be affected by meaningful work.

The physical environment was highlighted in both the work environment, *Having a meaningful work and a supportive work climate,* and the home environment, *Making your home your castle,* as essential for relaxation and positive interaction with others. Home and work interior designs structure social relationships and affect daily life activities [57] and should therefore be considered in health promotion work.

*Having a family and supporting network* is important to manage difficulties as it brings meaning and joy to life. The children, family and next of kin were the most important parts in life, and their well-being and health was seen as essential. Close and meaningful ties to others are an essential feature of what it means to be fully human. A major category of protective factors enabling individuals to overcome the life challenges derive from social relational ties [58]. Bourne et al. [59] found in their study that having time for family is the number one issue for employees. Organizations that embrace the whole individual must see to the importance of family-friendly employment practices, for example part-time work and control over work time [60]. To have a family gives energy, but it also takes energy and time. Work- and private life can be seen as a theoretical system in which the components of work and life interact and are dependent on each another [56] and a change in one system affects the other systems [61]. Managers would be wise to create work environments where success on the job reinforces success outside work and vice versa [59].

*Reflecting on life* was seen as a resource to balance work and private life in a satisfying way. To reflect on the meaning of life, to have the ability to set boundaries according to values and goals, the participants could easier balance everyday life. To decide what is most important makes it easier to decide how much time and energy to put into each domain and into each role in life. Ryff et al. [62] showed that women who reported higher levels of environmental mastery showed longer sleep duration and better quality of sleep as well as well-being. The participants in this study highlighted the importance of positive thinking, optimism and self- efficacy which is supported in Yarcheski et al. [63] study of important predictors of positive health. Furthermore, *Being healthy and taking care of yourself* was essential, for example to exercise and be in nature for recovery and energy mobilization. Ryan and Frederick’s [64] findings show that subjective vitality, defined as one’s conscious experience of possessing energy and aliveness, affects our health state. They also suggest that it is plausible to think that people high in subjective vitality may be more able to mobilize their resources to stave off disease processes or to be more actively participating in health promoting activities. Most studies of work-life balance literature have focused on factors at work and in the organization. There is a shortage of studies that focus on individuals and their internal resources and strategies. To explore differences in the way people balance work and private life is of importance [2,65].

### Methodological issues

The methodological aspects of credibility, dependability and transferability need to be considered for trustworthiness [35]. From a credibility perspective, triangulation was used. Individual interviews were conducted, analysed and the ‘preliminary’ themes were presented in focus group interviews for further discussions and validation. The participants were chosen by variation in age, marital status, number of children and age of children to increase the possibility of getting work-life balance illuminated from a variety of aspects. To establish credibility, the text was read several times, analysed separately and discussed by three researchers. The researchers’ background influences a study to some extent. The researchers had different backgrounds and pre-understanding about work-life balance, which was reflected and discussed to guard against researcher bias [36]. From a dependability perspective, the individual interviews were conducted in a short period of time. The participants answered the same main questions in the individual interviews. New insights on the phenomenon were discussed in the focus group interviews. The transferability is enhanced by descriptions of the participants, the data collection and analysis process. The findings of this study are illustrated by quotes and a selection of the analysis process is shown in Table 1. The findings of the study may be transferred to groups in similar work setting in Sweden, but it is the reader’s decision whether or not the findings are transferable to other contexts [35]. As this was a qualitative study, the aim was not to generalize but to gain a deeper understanding about the work-life balance phenomenon.

Limitations of the data should be noted. The participants in the study were women, no males chose to participate. Both men and women work in the departments of home help service but the majority is women. A gender perspective was not in focus in this study, but we are aware that the result would probably differ if the participants where a mix of women and men. Another limitation of the data was that the focus on resources, excluded perceptions on conflicts and demands, which may influence the work- life balance. Research on work-life balance with a salutogenic approach is narrow, and further studies are required to compare and complement resources that enhance work-life balance, for example studies that include males and are conducted in different work settings.

The comprehensive approach used in this study, includes both the organization and the individual and may create more favorable long-term outcomes than programs that focus solely on one factor [66]. The definition of work-life balance by Munn et al. [56] supports this approach; “Work-life balance is how an individual chooses to prioritize work, family, individual and community responsibilities. How one chooses to prioritize her or his work, family, individual and community responsibilities is in part influenced by the availability and knowledge of work-life initiatives as well as the organizational culture where it may or may not be acceptable to use [such] benefits”. (p.1).

### Implications

The result show that it seems favorable to create a supportive work environment which enhances both the individual and organizational resources. To reflect and communicate about values in life, find strategies and possibilities together may create an interface between the individual, the home and the organization to promote work-life balance. As work and private life change at different phases and transitions in life, the implications for work-life balance have to be flexible for the needs depending on the life situation. To create supportive environments for employees to manage their transitions in life would be a good and effective health-promoting strategy. In a study by Skinner et al. [42] the life course perspective is highlighted in order to understand and manage work-life interactions. Their findings indicate that the importance of diverse caring responsibilities over the life-cycle is recognized in organizational policies and practices. It is important to look at the individual as well as beyond the individual in health-related issues. Health of individuals is not created and lived in isolation, but is the result of an ongoing interaction with their socio-ecological environment [13,56]. It is of great interest that workplaces have strategies for work-life balance, offer part-time opportunities and provide a supportive work environment [67]. Furthermore it is important to realize that “Successful implementation of work-life balance programs requires more than a change in policy; it requires a change in institutional culture and mind-set” ([68], p.12). Managers in nursing contexts have a great challenge to create flexibility in the workplace, to see the whole person and maintain and strengthen recourses which enhance work-life balance and health of nurses. Employers, who invest in keeping their employees healthy and providing them the flexibility they require to meet their family needs, should be able to realize savings by reducing health costs, employee turnover, absenteeism and having a more committed workforce [69,70].

## Conclusion

The findings of this study show that organizations can work with a range of resources, like offering part-time work and a supportive work climate, in the workplace to promote work-life balance and health of nurses. Furthermore the findings show that individual actions like a healthy life style, positive relations and a positive home environment also may promote work-life balance. Interventions that address both the individual and the work setting seem essential and contribute to a variety of options for the organization and the employees. An intervention that gives possibilities to reflect on everyday life can be an important step in the workplace health promotion. Furthermore to organize the work so there can be time and energy for other important domains in life other than work.

By using a salutogenic approach, a main finding was that shift work can be a key resource when creating a sense of work-life balance. Further research is needed to gain a deeper understanding of this phenomenon.

## References

[1] Roberts K (2007). Work-life balance - the sources of the contemporary problem and the probable outcomes: a review and interpretation of the evidence. Employee Relat.

[2] Wayne J, Musisca N, Fleeson W (2004). Considering the role of personality in the work-family experience: relationships of the big five to work-family conflict and facilitation. J Vocat Behav.

[3] Haworth J, Lewis S (2005). Work, leisure and well-being. Br J Guid Counsell.

[4] Jacobs JA, Gerson K, Epstein CF, Kalleberg AL (2004). Understanding changes in american working time: a synthesis. Fighting For Time; Shifting Boundaries of Work and Social Life.

[5] Epstein CF, Kalleberg AL (2004). Fighting For Time; Shifting Boundaries of Work and Social Life.

[6] Voydanoff P (2005). Toward a conceptualization of perceived work-family fit and balance: a demands and resources approach. J Marriage Fam.

[7] Innstrand ST (2009). Gender-specific perceptions of four dimensions of the work/family interaction. J Career Assess.

[8] Emslie C, Hunt K (2009). Gender, work & organization: ‘Live to work’ or ‘work to live’? A qualitative study of gender and work-life balance among men and women in mid-life. J Fam Stud.

[9] Chang A, McDonald P, Burton P (2010). Methodological choices in work-life balance research 1987 to 2006: a critical review. Int J Hum Resour Manag.

[10] Wagman P, Björklund A, Håkansson C, Jacobsson C, Falkmer T (2011). Perceptions of life balance among a working population in Sweden. Qual Health Res.

[11] Casper WJ, Eby LT, Bordeaux C, Lockwood A, Lambert D (2007). A review of research methods in IO/OB work-family research. J Appl Psychol.

[12] Hammer L, Cullen J, Neal M, Sinclair R, Shafiro M (2005). The longitudinal effects of work-family conflict and positive spillover on depressive symptoms among dual-earner couples. J Occup Health Psychol.

[13] Bauer G, Davies JK, Pelikan J, Euphid Theory Working G, Euphid C (2006). The EUHPID health development model for the classification of public health indicators. Health Promot Int.

[14] Green J, Tones K (2010). Health Promotion: Planning and Strategies.

[15] Reid T, Quayle E (2008). A salutogenic perspective on occupational health. Irish J Psychol.

[16] Eriksson M, Lindström B (2008). A salutogenic interpretation of the Ottawa charter. Health Promot Int.

[17] Antonovsky A (1996). The salutogenic model as a theory to guide health promotion. Health Promot Int.

[18] Mackenbach JP, Van den Bos J, Joung IMA, Van de Mheen H, Stronks K (1994). The determents of excellent health: different from the determinants of Ill-health?. Int J Epidemiol.

[19] Seligman MEP (2008). Positive health. Appl Psychol.

[20] Whitehead D (2006). Workplace health promotion: the role and responsibility of health care managers. J Nurs Manag.

[21] Bringsén Å (2010). Taking care of Others – What’s in it for us? Exploring workplace-related health from a salutogenic perspektiv in a nursing context.

[22] Grosswald B (2004). The effects of shift work on family satisfaction. Families in Society - The Journal of Contemporary Human Services.

[23] Pressner BH (1994). Employment schedules among dual- earner spouses and the division of household labor by gender. American Sociological.

[24] Josefsson K, Sonde L, Winblad B, Robins Wahlin T-B (2007). Work situation of registered nurses in municipal elderly care in Sweden: a questionnaire survey. Int J Nurs Stud.

[25] Åkerstedt T, Ingre M, Eriksen C (2003). Work hour flexibility and the ability to sustain working life to retirement. Stress Research Reports.

[26] Horrigan JM, Lightfoot NE, Lariviere MAS, Jacklin K (2013). Evaluating and improving nurses’ health and quality of work life. Workplace Health & Safety.

[27] Steenbergen EF, Ellemers N (2009). Is managing the work-family interface worthwhile?: benefits for employee health and performance. J Organ Behav.

[28] Creswell JW (2007). Qualitative Inquiry & Research Design: Choosing Among Five Approaches.

[29] Patton QM (2002). Qualitative Research & Evaluations Metodhs.

[30] Heale R, Forbes D (2013). Understanding triangulation in research. Evid Based Nurs.

[31] Wallin OA (2013). Job satisfaction, strain and stress of conscience among nurse assistant working in residential care for older people.

[32] The official site of Sweden http://sweden.se/society/elderly-care-in-Sweden/#start (2014). Assessed 24 April 2014.

[33] Mishler EG (1986). Research Interviewing: Context and Narrative.

[34] Krueger RA, Casey MA (2009). Focus Groups: A Practical Guide for Applied Research.

[35] Graneheim UH, Lundman B (2004). Qualitative content analysis in nursing research: concepts, procedures and measures to achive trustworthiness. Nurse Educ Today.

[36] Burnad P (1991). A method of analysing interview transcripts in qualitative research. Nurse Educ Today.

[37] Szosland D (2010). Shift work and metabolic syndrome, diabetes mellitus and ischaemic heart disease. Int J Occup Med Environ Health.

[38] Di Lorenzo L, De Pergola G, Zocchetti C, L’Abbate N, Basso A, Pannacciulli N (2003). Effect of shift work on body mass index: results of a study performed in 319 glucose-tolerant men working in a Southern Italian industry. Int J Obes.

[39] Fenwick R, Tausig M, Epstein CF, Kalleberg AL (2004). The health and family-social consequences of shift work and schedule control: 1977 and 1997. Fighting For Time; Shifting Boundaries of Work and Social Life.

[40] Albertsen K, Rafnsdottir GL, Grimsmo A, Tomasson K, Kauppinen K (2008). Workhours and worklife balance. Scand J Work Environ Health.

[41] Yildirim D, Aycan Z (2008). Nurses’ work demands and work–family conflict: a questionnaire survey. Int J Nurs Stud.

[42] Skinner N, Elton J, Auer J, Pocock B (2014). Understanding and managing work-life interaction across the life course. Asia Pac J Hum Resour.

[43] Lindström B, Eriksson M: The Hitchhiker’s Guide to Salutogenesis: Salutogenic Pathways to health promotion.University West, Department of Nursing, Health and Culture; 2010.

[44] Carlson DS, Grzywacz JG, Zivnuska S (2009). Is work—family balance more than conflict and enrichment?. Human Relations.

[45] Rapoport B, Le Bourdais C (2008). Parental time and working schedules. J Popul Econ.

[46] Mills M, Täht K (2010). Nonstandard work schedules and partnership quality: quantitative and qualitative findings. J Marriage Fam.

[47] Pisarski A, Lawrence SA, Bohle P, Brook C (2008). Organizational influences on the work life conflict and health of shiftworkers. Appl Ergon.

[48] Pisarski A, Barbour J (2014). What roles do team climate, roster control, and work life conflict play in shiftworkers’ fatigue longitudinally?. Appl Ergon.

[49] Barnett RC, Hyde JS (2001). Women, men, work, and family: an expansionist theory. Am Psychol.

[50] Garey AI: Weaving Work and Motherhood. Philadelphia Temple University Press; 1999.

[51] Shagvaliyeva S, Yazdanifard R (2014). Impact of flexible working houers on work-life balance. Am J Ind Bus Manag.

[52] Jamieson LN, Williams LM, Lauder W, Dwyer T (2008). The ‘realities’ of part-time nursing: a grounded theory study. J Nurs Manag.

[53] Himmelweit S (2005). Making policymakers more gender aware: experiences and reflections from the Women’s budget group in the United Kingdom. J Women Polit Pol.

[54] Tracey C, Nicholl H (2007). The multifaceted influence of gender in career progress in nursing. J Nurs Manag.

[55] Nilsson P, Andersson I, Ejlertsson G, Troein M (2012). Workplace health resources based on sense of coherence theory. International Journal of Workplace Health Management.

[56] Munn SL (2013). Unveiling the work–life system: the influence of work–life balance on meaningful work. Adv Dev Hum Resour.

[57] Bell PA (1996). Environmental Psychology.

[58] Ryff DC, Singer B (2000). Interpersonal flourishing: a positive health agenda for the new millennium. Pers Soc Psychol.

[59] Bourne KA, Wilson F, Lester SW, Kickul J (2009). Embracing the whole individual: advantages of a dual-centric perspective of work and life. Business Horizons.

[60] Valcour M (2007). Work-based resources as moderators of the relationship between work hours and satisfaction with work-family balance. J Appl Psychol.

[61] Skyttner L (2001). General Systems Theory: Ideas & Applications.

[62] Ryff DC, Singer HB, Love DG (2004). Positiv health: connecting well-being with biology. The Royal Society.

[63] Yarcheski A, Mahon EN, Yarcheski JT, Cannella LB (2004). A meta-analysis of predictors of positive health practices. Journal of Nursing Scholarships.

[64] Ryan MR, Frederick C (1997). On energy, personality, and health: subjective vitality as a dynamic reflection of well-being. J Pers.

[65] Sumer HC, Knight PA (2001). How do people with different attachment styles balance work and family? A personality perspective on work-family linkage. J Appl Psychol.

[66] Noblet A, Lamontagne AD (2006). The role of workplace health promotion in addressing job stress. Health Promot Int.

[67] Silverstein M (2008). Meeting the challenges of an aging workforce. Am J Ind Med.

[68] Sullivan T (2014). Greedy institutions, overwork, and work-life balance. Sociol Inq.

[69] Halpern DF (2005). How time-flexible work policies can reduce stress, improve health, and save money. Stress Health.

[70] Pam A, Michael OD (2008). Positive effects of nonwork-to-work facilitation on well-being in work, family and personal domains. J Manag Psychol.

